# A qualitative examination of the implementation of a perinatal collaborative care program

**DOI:** 10.1017/S146342362200038X

**Published:** 2022-08-31

**Authors:** Bayley J. Taple, Shefali Haldar, S. Darius Tandon, Madhu Reddy, David C. Mohr, Emily S. Miller

**Affiliations:** 1 Center for Behavioral Intervention Technologies, Department of Preventive Medicine, Northwestern University Feinberg School of Medicine, Chicago, IL, USA; 2 Merck & Co., Inc, Boston, MA, USA; 3 Institute for Public Health and Medicine, Department of Medical Social Sciences, Northwestern University Feinberg School of Medicine, Chicago, IL, USA; 4 Department of Informatics, University of California, Irvine, CA, USA; 5 Department of Obstetrics and Gynecology, Warren Alpert Medical School of Brown University, Providence, RI, USA

**Keywords:** collaborative care, implementation strategies, perinatal, postpartum depression

## Abstract

**Aim:**

To identify implementation strategies for collaborative care (CC) that are successful in the context of perinatal care.

**Background:**

Perinatal depression is one of the most common complications of pregnancy and is associated with adverse maternal, obstetric, and neonatal outcomes. Although treating depressive symptoms reduces risks to mom and baby, barriers to accessing psychiatric treatment remain. CC has demonstrated benefit in primary care, expanding access, yet few studies have examined the implementation of CC in perinatal care which presents unique characteristics and challenges.

**Methods:**

We conducted qualitative interviews with 20 patients and 10 stakeholders from Collaborative Care Model for Perinatal Depression Support Services (COMPASS), a perinatal collaborative care (pCC) program implemented since 2017. We analyzed interview data by employing the Exploration, Preparation, Implementation, Sustainment (EPIS) framework to organize empirically selected implementation strategies from Expert Recommendations for Implementing Change (ERIC) to create a guide for the development of pCC programs.

**Findings:**

We identified 14 implementation strategies used in the implementation of COMPASS. Strategies were varied, cutting across ERIC domains (eg, plan, educate, finance) and across EPIS contexts (eg, inner context – characteristics of the pCC program). The majority of strategies were identified by patients and staff as facilitators of pCC implementation. In addition, findings show opportunities for improving the implementation strategies used, such as optimal dissemination of educational materials for obstetric clinicians. The implementation of COMPASS can serve as a model for the process of building a pCC program. The identified strategies can support the implementation of this evidence-based practice for addressing postpartum depression.

## Introduction

Perinatal depression, during pregnancy and up to 12 months postpartum, is common, with a new episode affecting over half a million women annually in the United States, with prevalence estimates ranging from ∼12% to 22% of perinatal individuals (Wisner *et al.*, 2013b; Woody *et al.*, 2017). Un- or under-treated perinatal depression can have devastating maternal consequences. One in five women with perinatal depression endorses suicidal ideation; suicide remains a leading cause of maternal mortality (Wisner *et al.*, 2013b; Shakespeare and Knight, 2015; Metz *et al.*, 2016; Mangla *et al.*, 2019). Not only does perinatal depression incur serious maternal risks but it has been associated with adverse obstetric and neonatal outcomes, including fetal growth restriction and preterm birth (Grote *et al.*, 2010; El Marroun *et al.*, 2012; Wisner *et al.*, 2013a; Staneva *et al.*, 2016). These complications influence neonatal morbidity and mortality and are an enormous economic burden to the healthcare system. Untreated perinatal depression can cause disruptions in infant attachment and infant brain architecture and physiologic dysregulations that can subsequently lead to learning or behavioral difficulties (Kingston *et al.*, 2012; Hoffman *et al.*, 2017; Aoyagi and Tsuchiya, 2019). As such, untreated perinatal depression is independently associated with long-term adverse neurodevelopmental consequences in offspring, with effects particularly pronounced in historically and socioeconomically excluded populations (Stein *et al.*, 2014).

Treatment of depression is associated with reduced perinatal risks. Compared to untreated depression, treatment with pharmacotherapy has been associated with a reduction in preterm birth, and treatment can mitigate the molecular dysregulation linked to adverse fetal programming (Hunter *et al.*, 2012; Kim *et al.*, 2015; Malm *et al.*, 2015). Additionally, psychotherapy, such as cognitive behavioral therapy (CBT), has shown reduction in perinatal depression, improved parent-child interactions, and reduced risk of child psychopathology (Cuijpers *et al.*, 2015; Letourneau *et al.*, 2017; Howard and Khalifeh, 2020). Adequate treatment of perinatal depression has critical public health implications for maternal and child health.

Despite the need for adequate treatment, meta-analytic data suggest that 95% to 97% of women with perinatal depression have under-treated symptoms (Cox *et al.*, 2016). Moreover, several recent studies have shown greater incidence of perinatal depression since the onset of the COVID-19 pandemic (Suwalska *et al.*, 2021). Barriers to effective perinatal depression treatment have been magnified during the pandemic. For instance, limited access to perinatal psychiatric care alongside increased demand for mental health care requires a greater focus on improving access to evidence-based mental health care for this population (Gordon and Borja, 2020).

Collaborative care (CC) was first developed to integrate mental health into an existing primary care structure. Mental health benefits are achieved through adherence to CC’s core principles (Huffman *et al.*, 2014). A care manager (CM) serves as the cornerstone of CC and facilitates initial treatment planning, brief behavioral care, longitudinal symptom monitoring, and implementation of specialist-informed stepped care recommendations (Miller *et al.*, 2020). Data from general primary care settings suggest that CC is both a clinically and cost-effective means to enhancing depression care and achieving depression remission (Reiss-Brennan *et al.*, 2010; Archer *et al.*, 2012; Jacob *et al.*, 2012; Katon *et al.*, 2012; Thota *et al.*, 2012; Holmes and Chang, 2022).

One small (*n* = 168) randomized trial evaluated CC in the perinatal context and showed improved perinatal depression outcomes for women randomized to perinatal collaborative care (pCC) compared to augmented usual care (Grote *et al.*, 2015). PCC led to a reduction in depression severity, greater adherence to depression care, and higher prevalence of depression remission, and pCC was cost-effective (Grote *et al.*, 2017). Despite these promising findings, further research is needed on pCC implementation to allow for scaling of pCC programs. A recent review on perinatal mental health indicated the need to evaluate and understand the implementation of mental health treatment programs in the context of perinatal care (Howard and Khalifeh, 2020).

Barriers remain to broader use of pCC because implementation strategies for CC have not been tailored to the unique perinatal context (Overbeck *et al.*, 2016). Perinatal care has distinct differences, compared with primary care, at the patient, clinician, and systems levels (Table 1) that require novel implementation strategies for the perinatal clinic setting. To bridge this gap, our goal is to provide empiric and actionable data to support implementation of the pCC model. We do this by identifying implementation strategies for CC applied in the context of perinatal care.

**Table 1:** Unique features of perinatal care

	Primary Care	Perinatal Care
Patient-level differences	Stigma of mental illness can be present	Stigma of mental illness also overlaid with stigma of being labeled a ‘bad’ mother or parent (Button *et al*., 2017)
	Concern/hesitation regarding use of psychotropic medications can be present	Concern specifically regarding how psychotropic medications may impact the baby through pregnancy and breastfeeding
Clinician-level differences	Longitudinal relationship with clinician	Multiple care transitions (obstetrician/midwife to pediatrician to primary care physician)
	Depression is more often seen within the scope of the clinician	Obstetricians feel inadequately trained to manage perinatal depression (LaRocco-Cockburn *et al*., 2003)
	Clinical care focused on the individual patient	Competing demands of patient and fetus/neonatal health care (Bowen *et al*., 2012)
	Multiple clinical visits	Postpartum care historically limited to one clinical visit (American College of Obstetrics & Gynecology, 2018)
System-level differences	Payment model	Bundled payment for pregnancy services without clear guidance pertaining to CC billing codes
	Lower prevalence rates of depression	Higher prevalence rates of depression (Wisner *et al.*, 2013b) that can overwhelm infrastructures

## Methods

### COMPASS Program

**Figure 1:**
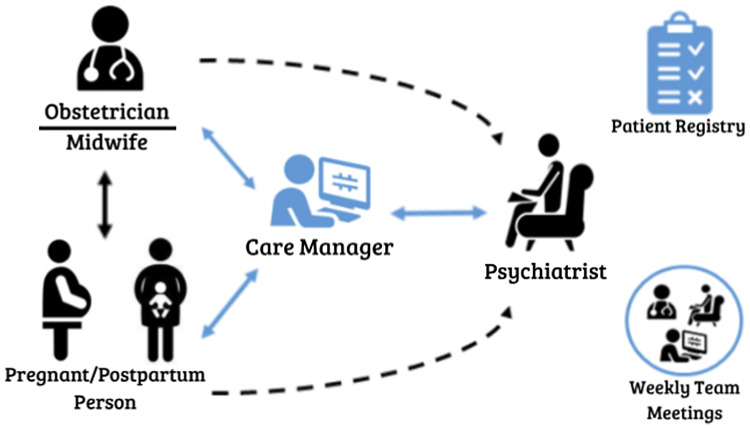
Diagram of the clinical workflow of the perinatal collaborative care (pCC) inner context. Novel aspects of clinical care are depicted in blue. Note, care managers may also serve as therapists when indicated in the patient’s care plan.

Collaborative Care Model for Perinatal Depression Support Services (COMPASS), a pCC program implemented within a large metropolitan medical center since January 2017, serves five affiliated outpatient obstetric clinics that collectively deliver approximately 3500 pregnant women annually. COMPASS follows the core principles of the CC model (Advancing Integrated Mental Health Solutions (AIMS) Center, 2020). Figure 1 demonstrates the clinical workflow and novel pathways introduced by pCC. Women are eligible for COMPASS services during pregnancy and up to 12 months postpartum. Obstetric clinicians give all women information about COMPASS at their first prenatal visit. Women with depression, by history or positive incident screen on the Patient Health Questionnaire (PHQ-9 ≥ 10 or ≥ 5 if criterial depressive symptoms are ≥2; Miller *et al.*, 2020), are referred by their obstetric clinician or self-referral. While a warm-handoff is the preferred referral mechanism to enhance continuity of care from obstetric clinicians to CMs, page, telephone, or electronic health record (EHR) messaging is utilized when physical distancing is required or when CMs are not immediately available.

After referral, the CM and patient connect in-person or via telemedicine to complete formal screening for psychiatric comorbidities and a thorough risk-assessment for suicidality and infant harm. Informed by this assessment, as recommended in the literature (Menear *et al.*, 2022), the CM engages in shared decision-making for an initial care plan with the patient and her obstetric clinician. The CM provides evidence-based psychotherapy for women with mild or moderate depression (Behavioral Activation (Martell *et al.*, 2013), CBT (Beck *et al*., 1979)). For women with moderate or severe depression or for whom psychotherapy is not chosen as a primary form of treatment, pharmacotherapy is initiated based on the evidence of whether the benefits outweigh the risks for the patient (Angelotta and Wisner, 2017). Some COMPASS patients receive both psychotherapy and pharmacotherapy, as appropriate. Previous studies have demonstrated that the combination of psychotherapy and medication treating depression may confer benefit (Khan *et al.*, 2012; Cuijpers *et al.*, 2014; Kamenov *et al.*, 2017). The CM refers women who require a psychiatric consultation (eg, complex pharmacotherapy history, bipolar disorder, suicidality) to a COMPASS perinatal psychiatrist, who assesses whether the patient is appropriate for pCC or if she requires a higher level of care with primary psychiatric treatment. CMs engage with patients until linkage to accessible and acceptable care is achieved.

All women in COMPASS are entered into a patient registry and counseled about the importance of depression symptom monitoring to gauge treatment response. Women are followed with automated email-based depression screens using the PHQ-9 (Spitzer *et al.*, 1999; Kroenke *et al.*, 2001). For women with active depressive symptoms (PHQ-9 ≥ 10 or ≥ 5 if positive for low mood and/or anhedonia), PHQ-9s are emailed every two weeks. Women in remission receive monthly PHQ-9 screens. If patients do not complete the screen within three days, the email is re-sent. If the screen remains uncompleted, the CM attempts contact via telephone; if a woman was symptomatic at her prior screen, the CM works with the obstetric clinician to elicit a response. For women receiving pharmacotherapy, data regarding medication adherence and side effects are also collected.

The multidisciplinary pCC team meets weekly to review new referrals and optimize initial care plans. Team meetings are a central component of CC (Miller *et al.*, 2020), where the team discusses women whose depression symptoms are not responding to treatment, and stepped care (optimization of pharmacotherapy, changes in frequency, or referral for psychotherapy) is recommended with input from the supervising psychiatrist. The CM communicates care plans to the patient and her obstetric clinician. The CM works with the obstetric clinician to ensure that mental health interventions are initiated within pCC. Using the AIMS protocol as a guide (AIMS Center, 2020), after 12 months postpartum, women are transitioned back to general primary care or community mental health services for ongoing depression monitoring and treatment. CMs engage with patients until successful transition.

### Implementation of COMPASS

**Figure 2:**
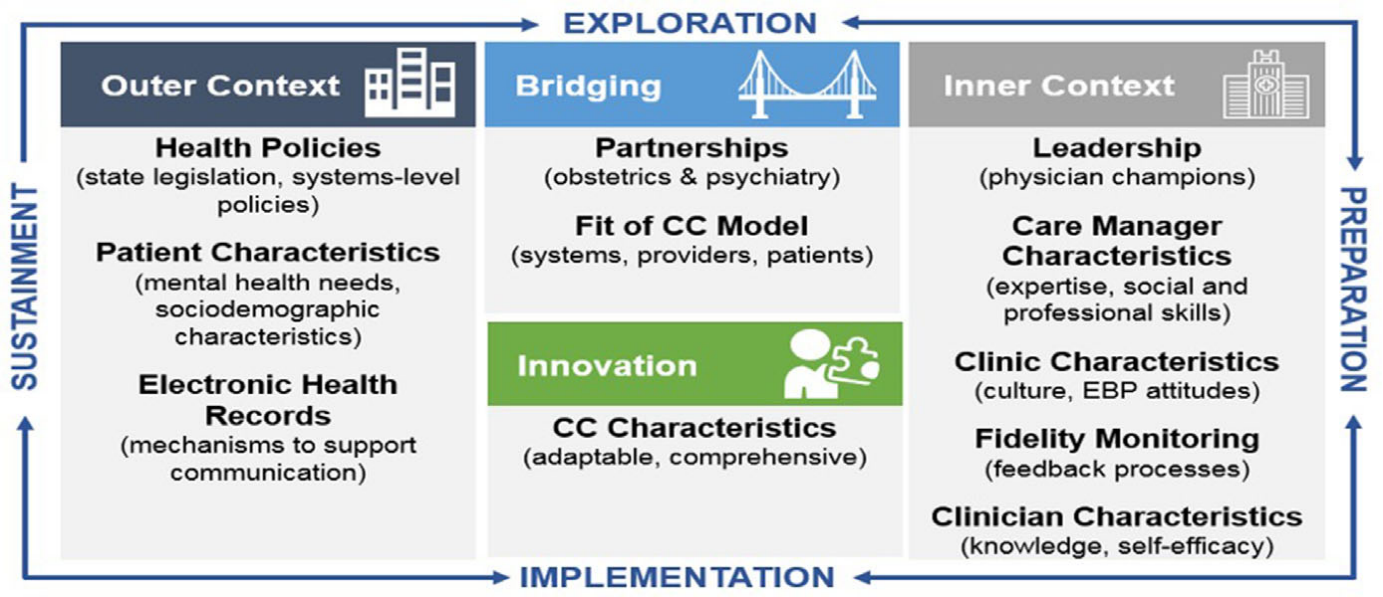
EPIS Process Model for Implementation of COMPASS

Implementation strategies are methods used to promote the translation of research to evidence-based interventions in clinical practice (Powell *et al.*, 2012; Powell *et al.*, 2015). The implementation strategies for COMPASS were selected empirically, based on the literature of CC in the primary care context (Gask *et al.*, 2010; Curran *et al.*, 2012; Eghaneyan *et al.*, 2014; Whitebird *et al.*, 2014; Overbeck *et al.*, 2016; Kwan *et al.*, 2017; Wood *et al.*, 2017). The Exploration, Preparation, Implementation, Sustainment (EPIS) process framework was used to systematically evaluate the contexts of care delivery (Figure 2; Aarons *et al.*, 2011; Moullin *et al.*, 2019). To enhance rigor and reproducibility, we codified the implementation strategies utilized in COMPASS with the Expert Recommendations for Implementing Change (ERIC) project, a compilation of 73 distinct implementation strategies (Powell *et al.*, 2015). We organized the implementation strategies within the EPIS framework and formed the basis of our codebook for qualitative analysis (Aarons *et al.*, 2011). The EPIS framework allows for clear understanding of the influences on implementation and to show how and in what contexts the implementation strategies fit for pCC. Notable characteristics of the outer context included state legislation mandated screening for postpartum depression and a hospital policy-mandated screening for perinatal depression at the first prenatal visits, in the third trimester, and postpartum. Additionally, the EHR system was homogeneous across outpatient and inpatient care, with a built-in mechanism to streamline provider-provider communication and expedite information sharing. The inner context included characteristics of the obstetrics practices as well as the CMs. The five obstetric clinics served by COMPASS were identified in the Exploration phase.

### Participants

We conducted a qualitative interview-based study with COMPASS patients and staff. All recruitment and study procedures were approved by the authors’ Institutional Review Board. Patients were eligible for this study if they were at least 18 years old, had been referred to COMPASS, and were pregnant or less than 12 months postpartum at the time of their interview. We followed a purposive sampling approach to recruit participants that reflected the demographic characteristics (ie, age, race, ethnicity, insurance) of the diverse population that COMPASS serves. A study team member contacted each eligible participant via phone or email, shared details about the study, and upon confirming interest in participation completed consent procedures. Approximately 15 people declined to participate due to lack of interest or could not be contacted in three attempts. A female postdoctoral fellow with extensive qualitative research experience, not directly affiliated with COMPASS to mitigate bias, conducted interviews at the time of contact or at a later date to accommodate patients’ availability.

For COMPASS stakeholders, we used a convenience sampling approach recruiting all staff associated with COMPASS. The COMPASS director established first contact via email, providing information about study details and goals. One hundred percent of staff participated in the study.

The study included 20 COMPASS patients (Table 2), 10 pregnant and 10 postpartum. Patients’ ages ranged from 24 to 37 years old (average 30.55 years). Twenty percent of patients identified as Black, 5% as Asian/Asian American, 5% as Native American, 50% as White, and 15% as ‘other’ or did not report their race. Thirty percent of patients reported Hispanic ethnicity. Regarding insurance, 65% of patients carried private insurance.

**Table 2:** Patient characteristics

	Patients
	*n* = 20
**Age**	
*Range*	24 – 37
*Mean (SD)*	30.55 (3.79)
**Race**	
White	10 (50%)
Black	5 (20%)
Asian/Asian American	1 (5%)
Native American/Alaskan Native	1 (5%)
Native Hawaiian/Pacific Islander	0 (0%)
Other	2 (10%)
Unknown	1 (5%)
**Ethnicity**	
Hispanic	6 (30%)
Not Hispanic	13 (65%)
Unknown	1 (5%)
**Insurance**	
Public	7 (35%)
Private	13 (65%)

Ten COMPASS stakeholders interviewed comprised three CMs, one OB/GYN nurse practitioner, one midwife, one OB/GYN resident, two psychiatrists, one therapist, and one clinical psychologist. All were women, with approximate age ranging from 25 to 55 years old. Seven stakeholders were White, two identified as Black, and one was Asian American. Experience working within COMPASS ranged from several months to three years.

### Study design, interview guide, and analyses

The study occurred from January to July 2020. One-on-one interviews with patients and stakeholders were completed via telephone (in-person with two staff). The goal of the patient interviews was to assess their overall experience with COMPASS and identify opportunities to improve upon and expand the scope of current services. Our semi-structured patient interview guide contained a range of questions about patients’ relationships with COMPASS healthcare providers, feedback on the services they used, and how they managed their mental health beyond COMPASS. The semi-structured clinician interview guide included questions about the clinician’s perception of their role within COMPASS, learning about pCC, and the impact to the clinic and their workflow. Interviews lasted 20–60 min, were audio recorded, and transcribed for analysis.

We followed a deductive thematic analysis approach to apply *a priori* codes that corresponded to the implementation strategies and EPIS framework (Hsieh and Shannon, 2005). We set recruitment goals for the interviews (*n* = 10 stakeholders and *n* = 20 perinatal patients stratified by pregnancy status [*n* = 10 pregnant, *n* = 10 postpartum]) to capture a diverse range of perspectives and to represent the COMPASS population. We were also guided by data saturation and continued recruitment until both thematic saturation and representation were achieved. We utilized *Atlas.ti* software to create categories of implementation strategies with supporting quotations. Two team members followed this analysis process independently. Consensus was achieved through regular meetings to discuss the codes, quotations, and coding consolidation until all transcripts had been analyzed and discrepancies adjudicated.

## Results

**Figure 3:**
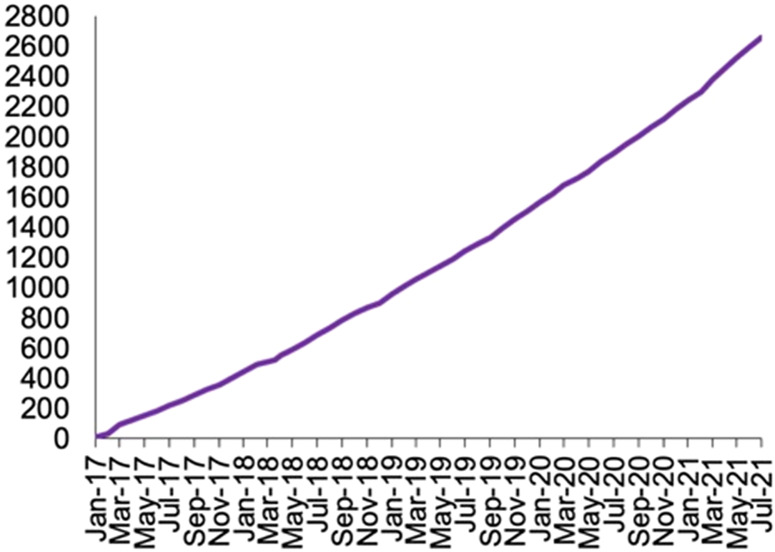
COMPASS referrals over time (January 2017 through July 2021)

Since its inception in January 2017, 30–60 women are referred to COMPASS monthly, with over 2200 referred in the first four years (Figure 3) and over 1700 (76%) of referred women enrolled. Screening for depression during pregnancy and postpartum increased after implementation of COMPASS (Miller *et al.*, 2021). Similarly, among those with a positive screen, obstetric clinician recommendation for treatment increased after implementation of COMPASS. We identify and describe implementation strategies, as defined by the ERIC Project (Powell *et al.*, 2015), derived from interviews, organized within the EPIS framework (Table 3; Aarons *et al.*, 2011). We observed variability in the types of implementation strategies used in COMPASS, such that they cut across the ERIC domains. This informs guidance pCC program implementation, such that a breadth of strategies is needed.

**Table 3:** Identified ERIC Strategies(Powell *et al.*, 2012, Powell *et al.*, 2015) for COMPASS Implementation Organized within the EPIS framework (Aarons *et al.*, 2011, Moullin *et al.*, 2019)

Inner Context	Bridging	Innovation	Outer Context
**Plan** 1. Assess for readiness and identify barriers and facilitators **Restructure** 2. Change physical structure and equipment 3. Facilitate relay of clinical data to providers 4. Create new clinical teams 5. Revise professional roles **Quality Management** 6. Use data warehousing techniques	**Plan** 7. Build a coalition 8. Identify and prepare champions 9. Intervene with patients/consumers to enhance uptake and adherence	**Educate** 10. Develop educational materials 11. Conduct educational meetings	**Attend to Policy Context** 12. Change liability laws **Finance** 13. Access new funding 14. Use other payment schemes

### Outer context

Outer context refers to characteristics of the broader environment outside of pCC that influence pCC, such as fiscal support.

#### Access new funding

COMPASS implementation was funded by a local philanthropic organization. Requests for proposals were solicited for clinical care programs that will advance women’s health initiatives in patient care, education, and community service. A core requirement for proposals was the potential for broad-based community impact on historically excluded communities and long-standing sustainability. To that end, the budget was designed to support integration of mental health care, with the vision that co-location could serve as a means for capacity building via facilitation of interdisciplinary conversations. One obstetric clinician (OC2) shared,

*‘I was actually super excited that [COMPASS program director] and company were able to get a grant and place [COMPASS] right on our floor for us to just walk over…I feel extremely well supported’.*

In addition, recognizing existing health disparities, designation of funds to support the direct delivery of care for those unable to access mental health care was prioritized. One midwife (OC1) mentioned,

*‘If patients are concerned about finances or they do not have mental health covered with their insurance, and that is a big consideration as I know that COMPASS … [has the] ability to see people even without coverage … I have never had anyone come back to me and say, ‘I was not able to see [COMPASS clinician] because of financial constraints’.*

Similarly, a perinatal psychiatrist (PSY1) stated,

*‘If you have a ton of money and have the ability to pay out of pocket, no matter what, you can get care. But a lot of patients don’t have that luxury [or] that opportunity. And worse… within [the] perinatal population’.*

Obstetric clinicians expressed being unlikely to engage in pCC when only a subsection of their patients would be eligible for participation. The complexity of understanding mental health benefits and difficulties with insurance alignment across departments or practices serves as barriers to partnership with mental health care. The understanding that all patients referred to COMPASS would receive care, regardless of insurance status or ability to pay, was an often-cited facilitator of utilization of the pCC model.

#### Change liability laws

Illinois Public Act 95-0469, Perinatal Mental Health Disorders Prevention and Treatment Act, was implemented on January 1, 2008, and requires that obstetric clinicians screen for postpartum depression. This local context, and early efforts to support implementation of this legislation, may have contributed to increased prevalence of completed postpartum depression screens before COMPASS implementation and a receptivity to change (Miller *et al.*, 2019). One obstetric nurse (OC2) illustrated the long-standing system of screening and documentation:

*‘When somebody has a new problem and obviously if there’s a mood disorder or … they have anxiety or depression … we add it. And now the mandate by the state of having a PHQ-9 started before COMPASS. [We monitor] OB patients at their new OB visit, at their 28-week visit, and at their postpartum visit, just to see how they were doing’.*

The state-wide attention and legislation regarding perinatal mental health assessments likely facilitated rapid implementation.

#### Use other payment schemes

A key component of COMPASS sustainability lies within the utilization of CC billing codes. The care coordination aspects of mental health care, including active patient reminders, surveillance of mood and anxiety screens, and communication regarding stepped care recommendations, have not been historically reimbursed. Centers for Medicare and Medicaid Services approved Medicare reimbursement for these services in October 2017 (Oquendo, 2016). As more commercial payers have followed suit, reimbursement for these services facilitates financial stability of CC programs and can enhance their dissemination (Miller *et al.*, 2020).

Importantly, billing codes for CC are time-based and linked to primary care, rather than mental health. Describing gaps in mental health coverage, patient (P02) stated, ‘*the [mental health] … referrals [out] that [COMPASS] sent, there wasn’t any for my insurance’.* An obstetric resident (OC3) explained the necessity of referring patients for mental health care:

*‘As OB-GYNs, we don’t have a lot of time to sit and address some of the mental health aspects of what a patient’s dealing with, right?…I can’t always sit and give this patient an hour of my time to [provide] mental health counseling. And I think that one of the great things about COMPASS…we’re giving patients a resource that they’re not having to worry about being financially responsible for to help them in the ways that we can’t’.*

As evidenced by the patient’s challenging experience finding mental health care outside of COMPASS and the obstetric clinician’s time constraints, pCC is a needed and valuable structure for providing and expanding access to perinatal mental health care.

### Inner context

The inner context includes characteristics and processes within the pCC program.

#### Assess for readiness and identify barriers and facilitators

Six months prior to COMPASS implementation, the COMPASS program director began attending meetings with each participating obstetric clinic to assess their capacity to support mental health care. An obstetric clinician (OC2) expressed initial feedback, ‘*I don’t have enough experience to deal with very significant psychiatric problems. It would scare me to death’.* Obstetric clinicians’ comfort with addressing mental health care evolved with longevity of the COMPASS program. OC3 said,

*‘Since I’ve been a resident, we’ve always had COMPASS. It’s been a part of the [OB ambulatory care] clinic since I started. I would say that overall, I feel pretty comfortable… screening for different issues. I talk to all my patients about whether or not they feel safe at home. I talk to people about sadness or depressed mood. And then if they answer… those questions, I can dive further in and be able to say, okay, I think this patient’s depressed based on a lack of sleep, or lack of interest, she’s feeling guilty, or no energy’.*

Despite the increase in comfort with mental health assessments and treatments, barriers to care remain. OC3 stated,

*‘I do not feel comfortable with providing counseling or resources [and when] patients are suicidal…I can’t say that I would know exactly how to address that. I would know how to say, ‘is this person immediately going to hurt themselves or hurt someone else’… but in terms of actually counseling to see how I could help them, not so much’.*

Identifying and respecting providers’ boundaries of comfort while building capacity requires close communication between leadership of the pCC program and the obstetric clinicians referring their patients to this unique model of care.

#### Change physical structure

Modifications of the physical space were identified as facilitators of implementation. The COMPASS psychologist (PSY3) described the process as an iterative assessment of

*‘What days were best. How that would interface with the OBGYN clinics. The [program director] identified a room for the coordinator and worked with her system there to ensure that that space could be devoted. There was work that was done with the front desk staff, she was sure that patient arrival and exit was appropriately documented and coordinated with the clinical care staff. There were discussions about how to coordinate communication between the COMPASS team…’*

Obstetric clinician (OC2) characterized pCC integration:

*‘I feel like we’re really giving the patient the one-stop-shopping. Lab is on our floor, the office is on our floor, ultrasound is on our floor, psych care is on our floor, social work is on our floor. So, it’s just I think great for our patient to know that we have the resources to help them’.*

Patient (P20) described the positive nature of COMPASS’ integration, ‘*mental health care that would work in tandem with my OB, which I felt like was good’.* However, patient (P05) noted that the COMPASS set up felt ‘*clinical…different from other therapists’ offices’.* Overall, both providers and patients highlighted the convenience and interdisciplinary care as a benefit of pCC.

#### Use data warehousing techniques to facilitate relay of clinical data to providers

A central component of pCC is the patient registry. Data warehousing storage supports completion of patient mood and anxiety screenings by providing a place to review incoming data and facilitating the relay of clinical data to providers. CM1 reflected,

*‘I use our COMPASS shared drive a lot. So, that’s a way for me to access daily reports…I get the most up-to-date data about a patient, so God forbid someone spikes up…we usually catch it pretty quickly’.*

Having a data infrastructure to support the registry was a key strategy facilitating adherence to the pCC model.

#### Create new clinical teams

Establishing the role of a CM into clinic workflow was a new addition to the obstetric clinical team. CMs are behavioral health professionals (eg, social workers) who are the lead contact person for the pCC model. CM responsibilities include coordinating treatment plans, assessing and monitoring patients’ symptoms, managing the patient registry, and leading communication with the psychiatry and obstetric teams regarding stepped care changes. Published CM caseload ratios emphasize the interplay between social determinants of mental health, psychiatric complexity, and medical complexity informs CM staffing ratios needed for pCC success (AIMS Center, 2017). The skills of the CM are cited as important enablers of pCC implementation including social skills (eg, ability to build relationships), engaging qualities, experience, and expertise (Curran *et al.*, 2012; Eghaneyan *et al.*, 2014; Whitebird *et al.*, 2014; Overbeck *et al.*, 2016). To develop the specific expertise required for COMPASS and to optimize scalability, CM training utilizes a curated program of freely available national resources (Table 4). Resources pertaining to perinatal mental health clinical guidelines are selected from the National Curriculum on Reproductive Psychiatry (National Curriculum in Reproductive Psychiatry, 2019).

**Table 4:** Recommended care manager training for COMPASS

Core Competencies	Training Resource	Module
Perinatal mental health care	National Curriculum on Reproductive Psychiatry (National Curriculum in Reproductive Psychiatry, 2019)	Depressive disorders module
		Anxiety disorders module
		Bipolar disorder module
		Trauma and post-traumatic stress disorder module
		Obsessive-compulsive disorder module
		Primary psychotic disorders module
		Infertility and loss module
Paternal mental health care	Fatherhood Research and Practice Network (Fatherhood Research and Practice Network, 2020)	Engaging Resident and non-resident fathers
Preventive health services utilization	ACOG (American College of Obstetrics & Gynecologists, 2016), AAP (American Academy of Pediatrics, 2020), and USPSTF (Siu *et al*., 2016) Guidelines	N/A
CC model overview	University of Washington AIMS Center (Advancing Integrated Mental Health Solutions (AIMS) Center, 2020)	Introduction to collaborative care
Care manager role		Introduction to care manager role
Management of a patient registry		Registry function in collaborative care
Communication with clinicians		Culture of primary care
Trauma-informed care	Substance Abuse and Mental Health Services Administration (Substance Abuse and Mental Health Services Administration, 2015)	Trauma-informed care
Health equity	Association of American Medical Colleges (Schaik *et al*., 2014)	Healthcare disparities

Participants highlighted the unique CM role within the pCC model as an important facilitator of implementation and utilization. Obstetric clinician (OC1) stated,

*‘[Care Managers] are the ones who are doing the initial outreach to the patient…usually that same day or within 24 hours. They are reaching out to see how the patient is doing… and assessing immediately to see what their needs are’.*

Their role, with designated ability to outreach with patients, created an immediate connection that facilitated patient engagement. Patient (P05) shared,

*‘So, when I reached out to multiple places, I ultimately decided to schedule an appointment through COMPASS. Even though I was thinking long-term, it would be the more inconvenient option. I decided to prioritize COMPASS because I felt like I had established a rapport and a connection with a person even before walking into the office’.*

#### Revise professional roles

In parallel with the creation of new clinical teams, pCC builds pathways of communication that promote capacity for screening and identifying mental health needs and for delivering mental health care. One psychiatrist (PSY1) stated,

*‘I feel like this is a good opportunity to increase access and also to ensure the kind of sustainability that comes with [CC], which means that you have to teach and train and help OBs launch because they’re gonna be the future of continuing this because there’s only so many psychiatrists’.*

PSY2 noted,

*‘I think also meeting the need of prescribing for COMPASS patients… really empowering [OBs] and educating them around the prescribing of psychiatric medication in pregnancy, particularly for more traditional straightforward depression/anxiety cases, trying to help bridge that gap where a psychiatrist may not always be available but being able to give OBs the resources and the guidance otherwise to do the prescribing for these patients’.*

By increasing the capacity of obstetric clinicians to address mental health issues, COMPASS has been able to maintain capacity of the psychiatrist to see new referrals or follow patients with more acute mental health needs. This revision of the role of the obstetric clinician is foundational to the success of a pCC program.

### Bridging

Bridging describes the processes used to build connections between the inner and outer contexts.

#### Build a coalition

CC is built upon the principle of interdisciplinary work. The psychologist (PSY3) reviewed the coalition building as follows:

*‘It took a lot of people from different disciplines, really earnestly trying to work together to put together a program focused on patient care… [there] were efforts to outline what the patient experience would look like, where that would happen. We did walkthroughs, identified the run, the logistics. The technical requirements for the [psychiatrists and therapist] …[and] how that was going to be coordinated across the weekday’.*

Coalition building extended beyond the outpatient setting itself. The team identified all points of potential patient contact and worked to ensure an awareness of the COMPASS program and care team. For example, PSY3 characterized the integration with the emergency department.

*‘There were setups of the suicide protocols that were also integrated into COMPASS, as well as cross-coordinated, extended into the ED. I met with ED psychiatry to ensure that they were clear if any of our patients in COMPASS, on site, needed to be handled like a psychiatric emergency. We had alignment …Whether that was during the weekday, during the clinical care hours, or off time’.*

This coalition is bridged via close communication between all members of each patient’s care team. Obstetric clinician (OC1) described this process:

*‘Anytime there is a touch point with the administration at COMPASS or a therapist or a psychiatrist, if anything has changed…we always get an email back, kind of like a consult note…through the [EHR]. And they outline that they have seen the patient and what is happening so we can follow up with them…’*

Close communication about patient care was a common thread of feedback about the success of the COMPASS program.

#### Identify and prepare champions

Obtaining buy-in from primary care clinicians has been identified as a critical, but challenging, aspect of CC implementation. Multiple qualitative analyses of CC implementation have demonstrated facilitation by clinician champions to be a key component of securing engagement of clinical staff (Overbeck *et al.*, 2016). Two specific roles of these champions include (1) ensuring coherence of the CC model among clinicians and (2) serving as local thought leaders pertaining to best clinical practices (Curran *et al.*, 2012; Sanchez and Adorno, 2013; Whitebird *et al.*, 2014). Considering these data, during the initial implementation meetings, the COMPASS program director and clinical liaison partnered with each obstetric clinic to identify an obstetric clinician champion. That person served as the point of contact with the COMPASS leadership team, to identify successes but also areas of ongoing need for improvement. Given the complexities of multidisciplinary care, such as pCC, having a champion able to understand the model and adapt the periphery to support implementation within the local context was identified by COMPASS leadership as instrumental to success.

### Innovation

Innovation within the EPIS framework designates the novel aspects of the pCC program.

#### Develop educational materials and conduct educational meetings

Care algorithms were created and tailored toward obstetric clinicians. These algorithms included ways to discuss depression screening with patients, interpretation of screen results, risk-risk decision-making for treatment of depression, and steps for prescribing and titrating psychopharmacology. CM3 shared,

*‘So, the [algorithms] for the OBs were disseminated at a Grand Rounds…[and] in all the workrooms. I’m sure most of them dissipated or disappeared. We also like to send it to the residents every time when they started and I think they would put them in the orientation workbook, too’.*

Creating and providing educational materials about perinatal mental health specifically targeted to obstetric clinicians was novel. While this was identified as an innovation, dissemination of education can be improved.

#### Intervene with patients/consumers to enhance uptake and adherence

CMs within COMPASS utilize multiple modalities to engage patients in mental health care, including the patient portal within the EHR, distribution of pamphlets for new obstetric patients, phone calls, or emails. While often effective, innovation within the realm of patient-provider communication was cited as a potential area of growth. CM2 outlined the use of EHR portal-based communication:

*‘[the portal] works really well for some people, but sometimes I will check [the portal], and people don’t respond to it…So, I personally would like to see a way that we could check in via text with people, or through an app, or something a little more updated…’*

Despite the lack of innovation with modalities of communication, patients expressed that the mere presence of a perinatal mental health program was enough to promote uptake. Patient (P13) said, ‘*It’s just nice to know that there’s a program that can help us get what we need and focus more on life’.* Patient (P17) expressed that she

*‘Liked the idea that it was a more holistic support. That I could just talk to someone about the options because before that I wasn’t really given options. I was just told, okay. Stop the meds to be safe, go see your therapist if you need to…I hoped to be able to maybe see a psychiatrist to prescribe medication. But also, just be able to talk to someone about how the mental health stuff intersected with pregnancy ‘cause no one had really talked to me about that before’.*

By filling the void that is currently present in most obstetric offices, COMPASS created a space for dialogue about mental health during pregnancy and postpartum.

## Discussion

The Institute of Medicine has identified a chasm between scientific knowledge and clinical practice (Institute of Medicine, 2005). The CC model is an exemplar of how to bridge this gap, with strong evidence of efficacy and effectiveness for depression in the primary care context (Coventry *et al.*, 2014; Sighinolfi *et al.*, 2014; Wozniak *et al.*, 2015; Huang *et al.*, 2017). However, implementation of CC is challenged by the complexity of the model itself, due to several active components and the need for multidisciplinary engagement (Figure 1). While an evidence base of implementation strategies for CC in the primary care context has emerged (Overbeck *et al.*, 2016; Kwan *et al.*, 2017), its utility in the perinatal context has been uncertain. Consequently, we used qualitative data to illustrate patient, obstetric provider, and mental health provider feedback on implementation strategies for COMPASS, a pCC program. Fourteen strategies were identified used in the implementation of COMPASS and were categorized using the EPIS framework to examine where and how these strategies were applied to better understand pCC implementation (Aarons *et al.*, 2011). Utilization of EPIS encourages the acknowledgment of differences between settings and adaptation of implementation strategies that may not align with local culture or context. Nevertheless, these strategies can serve as a scaffold upon which pCC can be implemented.

In the outer context for COMPASS, *accessing new funding*, *changing liability laws*, and *use of other payment schemes* were instrumental to implementation. While the former two may not be readily leveraged for all new pCC programs, CC billing codes can be applied in most contexts as both public and commercial insurance companies have agreed to reimburse for this evidence-based practice.

The inner context, on the other hand, refers to characteristics of the organization and can often be more easily modified. *Assessing for readiness and identifying barriers and facilitators*, *changing physical structure*, *using data warehousing techniques* to *facilitate relay of clinical data to providers*, *creating new clinical teams*, and *revising professional roles* were implementation strategies cited as foundational to COMPASS’ success. Each of these strategies has the advantage of being able to be adapted to the local healthcare system. For example, the *barriers and facilitators* to mental health care are unique not only to the healthcare system but also to each individual clinic. Though some barriers may remain, such as the physical structure of pCC clinic rooms feeling sterile compared to traditional outpatient mental health clinics. Implementation of pCC requires a willingness to learn from each site and adapt the system to their specific characteristics. *Creating new clinical teams* and *revising professional roles* are inter-related strategies for pCC – communication is an essential component of both, and both strategies also help address time constraints, a barrier identified in the literature (Aragones *et al.*, 2022) and by an OB in this study. The CM is a completely new healthcare role (Lattie *et al.*, 2021); ensuring that obstetric clinicians understand the CM role and that communication strategies are integrated between clinicians is important to emphasize. The identified components of bridging, including *building a coalition* and *identifying champions*, are some of the mechanisms to support communication around this new model of mental healthcare delivery, which is essential given that some OB providers continue to experience discomfort when assessing and discussing patients’ mental health concerns.

*Data warehousing* supports reflexive monitoring. Cited as a facilitator of sustained engagement in CC, reflexive monitoring is the opportunity to evaluate the impact of a new intervention (Overbeck *et al.*, 2016). It allows for effective treatment to target processes that are core to CC. Systematic feedback about patient progress back to the primary care clinician is a valued and validated method to promote engagement within CC (Curran *et al.*, 2012; Sanchez and Adorno, 2013; Eghaneyan *et al.*, 2014; Overbeck *et al.*, 2016) and has been cited a crucial element of pCC (Moore Simas *et al.*, 2018). Modalities to facilitate this communication, based on CM and obstetric clinician feedback, were developed for COMPASS. A CM-recorded trajectory of symptoms and current care plan are updated in each patient’s EHR ‘problem list’, easily accessible to all obstetric clinicians and integrated into their workflow. At times, patients do not respond to screeners and/or messages in their EHR portal, which can impact care coordination. All patient notes written by the CM or psychiatrist are routed in the EHR to the obstetric clinicians. These methods for communication of clinical data maintain obstetric clinician engagement as they feel ‘in the loop’ about their patient’s mental health care.

While two aspects of COMPASS’ implementation strategies were grouped under innovation, *developing educational materials* and *intervening with patients to enhance uptake and adherence*, optimally disseminating educational materials for obstetric clinicians and enhancing patient-provider communication remain opportunities for improvement. COMPASS materials are cited as valuable, but digitalization may improve distribution. In addition, pCC involves multiple team members, and optimizing efficient communication among obstetric clinicians, patients, and CMs remains a space for future growth and research.

### Limitations

Despite the many strengths of this study, there were some limitations. (1) We studied a single site, which may limit generalizability. This was partially mitigated by including five diverse obstetric offices within the medical center – a midwife practice, two OB/GYN clinics, a Maternal Fetal Medicine clinic, and an obstetric trainee clinic. (2) The sample size was small; however, previous literature has demonstrated that 10 people typically identify 84% to 95% of new concepts or problems (Faulkner, 2003; Turner-Bowker *et al.*, 2018). (3) The age of patients interviewed ranged from 24 to 37 years yet may not meet specific needs of adolescent birthing people or those of advanced maternal age.

## Conclusions

These limitations notwithstanding our findings provide a scaffold upon which clinicians and healthcare systems can begin to build a pCC program. These identified strategies, adapted to fit local resources and practices, can support the implementation of this evidence-based practice. Perinatal depression has an enormous impact on women and their infants; these implementation strategies can serve as a bridge to the necessary improvements in care to reduce the adverse impact of untreated depression.
